# A Rare Case of Contralateral Diaphragm Paralysis following Birth Injury with Brachial Plexus Palsy: A Case Report and Review of the Literature

**DOI:** 10.1155/2020/8844029

**Published:** 2020-11-12

**Authors:** Audra J. Reiter, Yazan K. Rizeq, Benjamin T. Many, Jonathan C. Vacek, Fizan Abdullah, Seth D. Goldstein

**Affiliations:** ^1^Ann and Robert H. Lurie Children's Hospital of Chicago, Division of Pediatric Surgery, Chicago, IL, USA; ^2^Department of Surgery, Feinberg School of Medicine, Northwestern University, Chicago, IL, USA

## Abstract

*Clinical History.* A 4.4 kg male was born to a 25-year-old, G2P1, nondiabetic woman at 39 and 5/7 weeks. Delivery was complicated by shoulder dystocia requiring forceps-assisted vaginal delivery, resulting in left arm Erb's palsy secondary to left brachial plexus injury. He was born with low muscle tone and bradycardia and subsequently required intubation for poor respiratory effort. He was extubated on day one of life but continued to be tachypneic and have borderline oxygen saturation, requiring intensive care. Chest radiographs demonstrated a progressive clearing of his lung fields, consistent with presumptively diagnosed meconium aspiration. However, a persistent elevation of the right hemidiaphragm was noted, and his tachypnea and increased work of breathing continued. Focused ultrasound of the diaphragm was performed, confirming decreased motion of the right hemidiaphragm. Following a multidisciplinary discussion, thoracoscopic right diaphragm plication was performed on the 33rd day of life. He was extubated postoperatively and subsequently weaned to room air with a notable decrease in tachypnea over 48 hours. He was discharged on postoperative day 12 and continues to thrive at 6 months of age without respiratory embarrassment. *Purpose*. Ipsilateral phrenic nerve injury with diaphragm paralysis from shoulder dystocia during vaginal delivery is a recognized phenomenon. Herein, we present a case of contralateral diaphragm paralysis in order to draw attention to the clinician that this discordance is possible. *Key Points.* According to Raimbault et al., clinical management of newborns who experience birth injury is a multidisciplinary effort. According to Fitting and Grassino, though most cases of phrenic nerve injuries are ipsilateral to shoulder dystocia brachial plexus palsy, contralateral occurrence is possible and should be considered. According to Waters, diaphragm plication is a safe and effective operation.

## 1. Introduction

Nerve traction injury from shoulder dystocia during vaginal delivery is a recognized phenomenon, most often resulting in a brachial plexus, or Erb's palsy. In rare instances, the insult is sufficiently severe that there is a concomitant ipsilateral phrenic nerve injury (PNI) with diaphragm paralysis that causes respiratory embarrassment or failure. Here, we present a case of diaphragm paralysis contralateral to the brachial plexus injury, which, to our knowledge, is only one of the two reported occurrences in the literature [1]. This report also aims to review the existing literature on diaphragm paralysis from birth trauma.

## 2. Case Report

A 4.4 kg male was born to a 25-year-old G2P0 at 39 and 5/7 weeks, complicated by shoulder dystocia, requiring forceps-assisted vaginal delivery. Of note, the amniotic fluid was stained with thick meconium at delivery. He was born with low tone, heart rate less than 50 BPM, and required positive pressure ventilation with APGARS of 1, 6, and 8 at one, five, and ten minutes, respectively. Within fifteen minutes of birth, he required endotracheal intubation for poor respiratory effort. He was extubated on day one of life but continued to be hypoxic and tachypneic, which were attributed to presumed meconium aspiration. He was also noted at this time to hold his left upper extremity in adduction and internal rotation with minimal movement. His left upper extremity exam was significant for persistent weakness in wrist extension/finger extension and shoulder adduction, consistent with an Erb's palsy. There were no obvious deformities of his clavicle or humerus, and his pulses were intact. His right arm was completely unremarkable with normal strength and tone.

**Figure 1:**
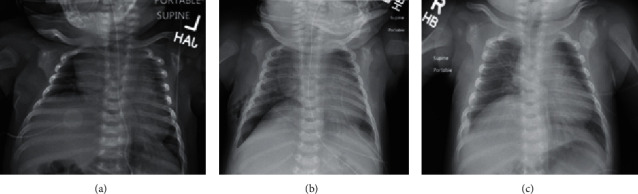
(a) Chest radiograph prior to surgery showing elevation of right hemidiaphragm. (b) Chest radiograph immediately after surgery demonstrating expansion of the right lung field with small basilar pneumothorax and minimal subcutaneous emphysema. (c) Chest radiograph one-month postoperatively demonstrating continued increased right hemithorax volumes with excellent lung expansion.

Subsequently, with assistance from occupational therapists, the infant began to recover strength and spontaneous movement of the left arm. However, he continued to require cardiorespiratory monitoring, critical care management, and oxygen via high flow nasal cannula for the next 21 days. During his critical care stay, radiographs demonstrated a progressive clearing of his lung fields as well as persistent elevation of his right hemidiaphragm (Figure 1(a)). It eventually became clear that his need for oxygen supplementation was exceeding the expected course of meconium aspiration. Ultrasonography of the diaphragm was performed, demonstrating decreased motion of the right hemidiaphragm and normal movement of the left hemidiaphragm. Due to the discordance in laterality between the brachial plexus injury and diaphragm dysfunction, presumed to be from an occult contralateral phrenic nerve injury, a thoracic MRI was obtained to rule out other unexpected pathology, such as soft tissue injury or any intrathoracic pathology like a soft tissue mass causing compression on the cervical nerve roots or phrenic nerve, but was only notable for findings associated with the known left brachial plexopathy.

**Figure 2:**
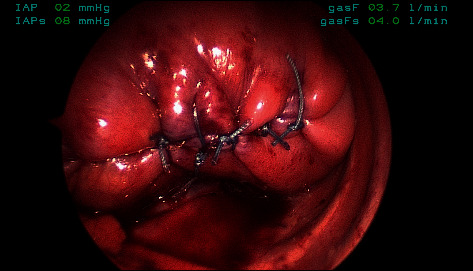
Image of diaphragm flattened following vertical row of sutures, slight dome shape is noted, however, not under any remarkable tension.

Following a multidisciplinary discussion, thoracoscopic right diaphragm plication was chosen as the management strategy. This decision was influenced by the fact that our patient was requiring high-flow nasal cannula and was unable to be weaned over multiple weeks. Originally, we thought the respiratory distress may have been due to meconium aspiration, but after there was no improvement over the expected time period, this moved lower on our differential. The continued need for hospital care in an otherwise well baby ultimately indicated surgical plication. He was taken to the operating room and positioned in left lateral decubitus position after induction of general anesthesia and endotracheal intubation. Using a 4 mm thoracoscope and two 3 mm instrument trocars, a flaccid right hemidiaphragm with bulging central tendon was noted, then linearly plicated, and flattened. A self-locking suture was used in a running fashion to imbricate the central tendon down into the abdomen, bringing the muscular edges together and beginning the plication. This method also served to reduce the tension on our final suture line. After this was completed, with care to keep the neurovascular bundle out of harm's way, a second row of vertical mattress sutures was placed to further flatten the hemidiaphragm. At the completion of this step, the diaphragm was noted to be reasonably flat with only the slightest dome shape, though not under any remarkable tension at rest (Figure 2). He tolerated the procedure without complication and was extubated before returning to the neonatal intensive care unit (NICU), where his respiratory rate was immediately noted to be lower than preoperatively. His initial postoperative radiograph showed a flattened right hemidiaphragm, increased aeration of the right lung, and a small basilar right pneumothorax that did not require chest tube placement. By the second postoperative day, he was off supplemental oxygen and tolerating an oral feeding regimen. He was transitioned off-tube feeds over the next 10 days and was discharged home on postoperative day 12 (Figure 1(b)).

## 3. Discussion

Phrenic nerve stretch injury leading to diaphragm paralysis in newborns is a known complication of shoulder dystocia. The phrenic nerves originate bilaterally from the C3 to C5 vertebral nerve roots in the neck, run along the ventral surface of the anterior scalene muscle, and are covered by the sternocleidomastoid. In the thorax, they cross the apex of their respective ipsilateral pleura to course caudally on the lateral aspects of the pericardium until they arborize onto their respective hemidiaphragms [2–4]. The phrenic nerves supply the only motor innervation to the diaphragm; thus, injury during delivery can result in respiratory distress due to paralysis and ultimately a bulging eventration of the ipsilateral diaphragm. This is a distinct entity from iatrogenic PNI following correction of congenital cardiac conditions, as well as from congenital eventration. In the latter, the central tendon is attenuated and bulging, but the muscular portion is innervated and contracted. First reported in 1902 by a German obstetrician, Bernhard Naunyn, there have been fewer than 100 reported cases in the literature, the largest series comprising 18 infants (Table 1) [5].

The most common comorbidity associated with PNI is brachial plexus palsy (BPP). BPP has been previously reported to occur in 1–4 per 1000 live births [6]. Remarkably, an estimated 66–92% of these infants spontaneously recover before two months of life [5, 7–12]. Though only 2–5% of infants with dystocia-related BPP have PNI, a majority of PNI will have concomitant BPP [13]. To date, PNI, if present, has been almost exclusively reported on the ipsilateral side of BPP.

Longitudinal studies have assessed risk factors in infants for developing PNI during delivery, such as shoulder dystocia, large for gestational age, maternal diabetes, and use of forceps, breech, or vacuum delivery [13, 14]. The aforementioned risk factors were found to be associated with a higher risk of PNI during the perinatal period, but they were only found to be predictive in 10–19% of cases. In addition, PNI was observed in cases where the mother underwent cesarean section, and in 37–47% of vaginal deliveries without shoulder dystocia, indicating that some PNI may be due to an inutero mechanism [7]. The incidence of PNI from birth trauma is estimated to be 1 per 15,000–30,000 live births, and mortality is estimated at 10–15% [8, 11, 15]. Management of these patients has not been standardized, but the majority of patients requires mechanical ventilation, oxygen, and nasogastric tube feeding to ensure weight gain. Clinicians generally consider the following options: (1) immediate diaphragm plication, (2) delayed plication following a period of observation for spontaneous recovery, and (3) a supportive nonoperative approach with prolonged mechanical ventilation or appropriate respiratory support [16, 17].

Historically, thoracotomy with plication was the first reported surgical correction for PNI with diaphragm paralysis. Although this operation may offer benefits, the morbidity and associated spinal scoliosis made thoracoscopy an attractive technique. A thoracoscopic approach is particularly beneficial for a right-sided diaphragm plication due to the presence of the liver under the right hemidiaphragm. The liver helps to protect from bowel injury when suturing the diaphragm from an intrathoracic approach, but also makes a laparoscopic approach more difficult as it blocks access to the right hemidiaphragm when approaching intraabdominally [18–20]. Another option for repair is a laparoscopic approach, theorizing that patients tolerate laparoscopy better than thoracoscopy, as well as the surgeon having more working room in the abdomen as compared to the thorax. Laparoscopic diaphragm plication is well-tolerated in the pediatric population, so operative approach should be determined on a case-by-case basis considering the patient history, laterality, and the experience and comfort level of the surgeon [21, 22].

Indication and timing for thoracoscopic diaphragm plication (TDP) are both controversial and should be addressed in a multidisciplinary fashion. The spectrum of PNI extends from minor traction palsy with rapid recovery to more severe injuries leading to chronic respiratory failure [5, 7–11]. Thus, the benefits of plication and subsequent improvements in ventilation must be weighed against the perioperative morbidity, especially in the context of reports of spontaneous recovery without intervention [9, 11, 12, 19–21, 23]. However, the time course of spontaneous recovery is variable, from 7 days to 6 months, often requiring resource-intensive hospitalization with respiratory monitoring, positive pressure assistance, or even mechanical ventilation [5, 7–12]. TDP is generally associated with a shorter length of stay as compared to open diaphragm plication (DP) and has a low reported complication rate. Kozlov and Novozhilov found an overall average length of stay of 9.88 days, 3.23 days in the NICU, with the thoracoscopic approach compared to 13.06 days, 5.89 days in the NICU, with the open approach [24].

With respect to timing, prior authors have been split between advocating either early (<30 days) or late (>30 days) DP [25]. One of the larger reports by De Vries et al. reported 18 patients with PNI and DP, where 13 plications were performed. The majority of those cases (12 of 13) were performed after 30 days of life. The authors advocated observation for the first 30 days of life to ensure the baby would not spontaneously recover. They found that none of their patients recovered after 30 days; therefore, the decision to operate should not be delayed any longer than a month [1]. In contrast, for patients with PNI and DP, Stramrood et al. performed 80% of their surgical plications before 30 days of life and found earlier plications yielded better respiratory outcomes than plications after 30 days [8]. More recently, Ahamdpour-Kacho et al., and Garge performed plications before 30 days of life and were able to wean their patients off ventilator support earlier, minimizing the morbidity associated with long-term mechanical ventilation. [16, 23]. Based on our literature review, the earliest reported surgical intervention was at 10 days, the latest was 15 months, and the median was 35 days after diagnosis.

In our case, we operated on day 33 of life, which would be considered a late plication, though delay in diagnosis played a role due to the unusual presentation of the diaphragm paralysis contralateral to the BPP. The postoperative recovery was remarkable, with immediate extubation and improvement in respiratory status. On this basis, we tend to recommend consideration of early plication in instances in which an earlier diagnosis is made. In an era prior to thoracoscopy, the morbidity of surgical repair would have been perceived as a higher barrier to surgical intervention for these infants. However, with modern anesthetics and a minimally invasive approach, we see no advantage to delaying repair once diaphragm paralysis has been established. This only adds to the overall and postoperative hospital length of stay, which we recently demonstrated in a large national database [25].

Our literature review revealed only one other case report of contralateral hemidiaphragm paralysis by Pegu et al. In that case, the infant was also large for gestational age at 4.4 kg, requiring a vacuum-assisted delivery. The baby was noted to have a left-sided Erb's palsy at birth and developed respiratory distress shortly after birth with a chest radiograph that showed right hemidiaphragm elevation. In contrast to our case, an initial ultrasound was inconclusive for diaphragm paralysis, but a repeat study was consistent with diaphragm paralysis, so the baby underwent TDP at 2 months of age. The infant required supplemental oxygen until 6 months of age, but was able to tolerate oral feeds without tube feed supplementation [31]. In both cases, diagnosis was challenging due to the fact that the diaphragm paralysis was contralateral to the side of Erb's palsy. Our assumption is that the intrapartum mechanical forces on the neck can lead to both the brachial plexus neuropathy as well as the contralateral phrenic nerve injury. This is conceivably the result of multiple rotation or side flexion events. In our case, the baby was operated at one month of age as compared to 2 months of age, which led to the ability to wean oxygen much faster. By postoperative day 2, our patient was off supplemental oxygen and tolerating oral feeds, but it took the baby, in Pegu et al.'s study, 6 months to completely wean from oxygen.

Making the diagnosis of PNI is the first barrier to avoiding delays in care for these infants. With a traditional presentation of respiratory distress in the setting of shoulder dystocia and BPP, a chest radiograph should be obtained. If an elevated hemidiaphragm is demonstrated, then the diagnosis of PNI is suspected. This will most often be noted ipsilateral to the BPP, but our case is meant to alert clinicians to maintain an index of suspicion for contralateral deficits as well. Ultrasound can confirm presence or absence of diaphragm excursion, the extent of excursion, any differences between the two hemidiaphragms, as well as any paradoxical movement. After imaging is obtained and there is a suspicion for PNI, a multidisciplinary discussion regarding surgical plication should commence.

## 4. Conclusion

Having a high index of suspicion for phrenic nerve injury resulting in diaphragm paralysis, related to a contralateral brachial plexus injury during birth, is very important to prevent a delay in diagnosis. Monitoring the baby closely for spontaneous recovery is important in PNI, but discussion about diaphragm plication should start early. With the ability to perform minimally invasive surgery, we favor early plication over late plication, which we believe decreases the morbidity associated with prolonged ventilation, requirement for nasogastric tube feeding supplementation, and length of hospital stay.

## Figures and Tables

**Table 1:** Pertinent cases of newborns who suffered birth trauma and PNI with diaphragm paresis [1, 8–11, 13, 15, 16, 23, 24].

Study	Patients	Sex	Phrenic nerve injury side (R/L/B)	Laterality paralysis(R/L/B)	Birth injury	Radiology	Day of plication following diagnosis	Days/months following surgery to wean off MV
Bowman et al. [26]	1	Male	B	B	Breech presentation	X-ray	140	14 days
Jawad et al. [10]	3	1 male 2 females	3R R,R	1B, 2R	1 breech 2 p rolonged labour	X-ray US	28, 42, NP	NR
Commare et al. [27]	3	1 male 2 females	3 B	3 B	3 breech presentation	X-ray US	NP 2, 60	4mos
De vries et al. [1]	18	12 males 6 females	4L, 14R	4L, 14R	11 breech presentation 4 v acuum delivery 2 e xcessive b irth weight 1 f orceps	X-ray US	10, 32, 33 22, 45, 46, 62, 86, 105, 154, 189, 210, 292, NR, NR, NR, NR, NR	“All patients who underwent plication were discontinued within a few days after operation”
Shmizu et al. [15]	1	Male	B	B	Breech presentation	X-ray US	45	8 days
Karabiber et al. [28]	1	Male	L	L	Shoulder dystocia Breech presentation	X-ray US	56	5 days
Stramrood et al. [8]	14	7 males 7 females	10R, 3L 1B	10R, 3L 1B	4 breech presentation 3 cephalic 4 breech presentation 2 cephalic 1 transverse	X-ray US	10, 12, 13, 15, 17, 20 (2), 23, 50, 51, NP 3	NR (4), 2 days (2), 3 days (2), 4 days, 6 days (2), 8 days, 17 days, 58 days
Ahamdpour-kacho et al. [16]	1	Male	R	R	Breech presentation	X-ray US	<30	3 days
Heritier et al. [29]	1	Female	L	L	Forceps	X-ray US		3 days
Shiohama et al. [30]	2	1 male 1 female	R B	R B	Breech presentation Uneventful	X-ray US	>30 15 months	NR (2)
Bowerson et al. [11]	4	3 males 1 female	2B, 2R	1B, 3R, 1B, 3R	2 shoulder dystocia Forceps Breech	X-ray US	30, 35, 60, NR	3 days, 5 days, 13 days, NR
Murty et al. [9]	1	Female	R	R	Breech presentation	X-ray US	NP	7 days
Garge et al. [23]	1	Male	R	R	Breech presentation	X-ray US	17	3 days
Pegu et al. [31]	1	Female	L	R	Vacuum delivery Large for gestational age	X-ray US	60	2 months

NP: no plication, NR: not reported.

## Data Availability

The data used to support the findings of this study are available from the corresponding author upon request.
